# Machine Learning- and Deep Learning-Based Myoelectric Control System for Upper Limb Rehabilitation Utilizing EEG and EMG Signals: A Systematic Review

**DOI:** 10.3390/bioengineering12020144

**Published:** 2025-02-03

**Authors:** Tala Zaim, Sara Abdel-Hadi, Rana Mahmoud, Amith Khandakar, Seyed Mehdi Rakhtala, Muhammad E. H. Chowdhury

**Affiliations:** 1 Department of Electrical Engineering, Qatar University, Doha 2713, Qatar; tz2104202@qu.edu.qa (T.Z.); sa2001791@qu.edu.qa (S.A.-H.); rm2005272@qu.edu.qa (R.M.); amitk@qu.edu.qa (A.K.); 2 School of Engineering, University of the West of England, Bristol BS16 1QY, UK

**Keywords:** EMG, EEG, machine learning, deep learning, upper limb, arm, disability, disabilities

## Abstract

Upper limb disabilities, often caused by conditions such as stroke or neurological disorders, severely limit an individual’s ability to perform essential daily tasks, leading to a significant reduction in quality of life. The development of effective rehabilitation technologies is crucial to restoring motor function and improving patient outcomes. This systematic review examines the application of machine learning and deep learning techniques in myoelectric-controlled systems for upper limb rehabilitation, focusing on the use of electroencephalography and electromyography signals. By integrating non-invasive signal acquisition methods with advanced computational models, the review highlights how these technologies can enhance the accuracy and efficiency of rehabilitation devices. A comprehensive search of literature published between January 2015 and July 2024 led to the selection of fourteen studies that met the inclusion criteria. These studies showcase various approaches in decoding motor intentions and controlling assistive devices, with models such as Long Short-Term Memory Networks, Support Vector Machines, and Convolutional Neural Networks showing notable improvements in control precision. However, challenges remain in terms of model robustness, computational complexity, and real-time applicability. This systematic review aims to provide researchers with a deeper understanding of the current advancements and challenges in this field, guiding future research efforts to overcome these barriers and facilitate the transition of these technologies from experimental settings to practical, real-world applications.

## 1. Introduction

Motor impairments frequently affect individuals with neuromuscular conditions, including stroke, spinal cord injury, and muscular dystrophy, as well as those with neurodegenerative diseases like multiple sclerosis and amyotrophic lateral sclerosis [1,2]. These disabilities can significantly hinder an individual’s ability to perform essential tasks including mobility and self-care, thereby impacting their overall quality of life [3]. This highlights the critical need for rehabilitation to reduce adverse outcomes, optimize motor functioning, and improve patient well-being [4]. Patients often require continuous assistance with even the most basic tasks [5]. In such cases, wearable robots, commonly referred to as exoskeletons, provide continuous assistance and support [6]. Myoelectric-controlled systems used in upper limb exoskeletons primarily rely on surface electromyography (sEMG) signals to detect muscle activity and can also be enhanced by incorporating electroencephalography (EEG) signals for more comprehensive control [7].

Invasive signals involve the direct collection of neural data from within the brain through surgically implanted electrodes, which can capture electrical signals from neurons with high precision [8]. Techniques like intracortical microelectrode arrays and electrocorticography (ECoG) are commonly used for this purpose [9]. In contrast, non-invasive signals are obtained without surgery, using external sensors positioned on the scalp or other areas of the body [10]. While invasive methods offer higher spatial resolution and more detailed neural data, non-invasive methods are often preferred due to their safety, ease of use, and reduced risk of complications [11]. Non-invasive methods, like EEG and electromyography (EMG), are particularly advantageous in clinical settings and for long-term monitoring, where minimizing patient risk and discomfort is crucial [12].

Surface electromyography (sEMG) and EEG are key non-invasive techniques utilized in the development of myoelectric-controlled systems for rehabilitation [13]. sEMG records electrical potentials from skeletal muscles during muscle fiber contraction or relaxation [14], providing valuable data for controlling devices such as upper limb exoskeletons [15]. On the other hand, EEG measures brain activity and might be utilized to control external devices, such as robots, straight through brain signals [16]. This technology offers enhanced mobility and independence for individuals with physical disabilities [17]. Both sEMG and EEG signals are crucial for developing advanced rehabilitation systems that can detect motion intention and improve human–robot interactions [18]. sEMG signals enable the detection of motion intention before physical movement [19] and are often integrated with kinematics data retrieved from specialized sensors such as inertial measurement unit (IMU) sensors [20], while EEG provides insights into brain activity patterns that can be used to control robotic systems [21]. The integration of these signals with machine learning (ML) or deep learning (DL) models allows a comprehensive understanding of muscle and brain activities, improving the accuracy of control and feedback in rehabilitation devices [22]. Fu et al. (2022) [23] performed a comprehensive systematic review of the advancements in myoelectric-controlled systems used in upper limb wearable robotic exoskeletons and exosuits. The review underscores the significance of EMG signals in detecting motion intention before physical movement. Contrasted to other controlled systems, a key strength of myoelectric control is its ability to detect the motion intention of the user promptly by utilizing electromechanical delay; this approach can identify the onset of motion intention approximately 50–100 milliseconds before the actual physical movement occurs [24,25], enabling more adaptive and intelligent interactions between humans and upper limb wearable robotic exoskeletons [26]. The review also emphasizes the importance of improving the robustness of ML-based controlled systems and developing human-centered approaches in future research. The paper also discusses how machine learning techniques have been utilized in upper limb exoskeletons to anticipate movement patterns from EMG signals through different methods. It highlights that these myoelectric control systems utilize statistical features extracted from preprocessed EMG signals, like mean absolute value (MAV), wavelength (WL), and root mean square (RMS) [27]. These features are subsequently utilized as inputs for different machine learning models, enabling tasks such as detecting movement types via classification models [28,29], estimating joint angles or torque using regression models [30,31], and reducing muscle effort during assistance through reinforcement learning [32]. Aljalal et al. (2020) [33] provided a thorough review of brain-controlled robotics, particularly focusing on non-invasive brain–computer interface (BCI) systems that utilize EEG signals. This review explores the promising potential of EEG technology to directly control external devices, such as robots, through brain activity. This capability significantly enhances mobility and independence for individuals with physical disabilities. The review also emphasizes the importance of integrating various machine learning models, such as Artificial Neural Networks (ANNs), Linear Discriminant Analysis (LDA), and Support Vector Machines (SVMs), in accurately interpreting EEG data for effective robotic control system.

This study aims to thoroughly review and analyze current ML and DL techniques applied to EMG and EEG signal analysis within the realm of rehabilitation for patients with disabilities. It seeks to identify and compare the strengths and limitations of these models, evaluate their effectiveness in enhancing rehabilitation efficiency and accuracy, examine the significance of integrating EMG and EEG signals to improve patient recovery outcomes, and highlight evolving trends and future directions in this field.

Several advanced rehabilitation solutions have been developed to assist individuals with upper limb impairments, utilizing cutting-edge technologies to enhance motor and cognitive functions. Myro is an interactive, sensor-based device that detects touch, force, and object movement, enabling task-oriented therapy with real objects and gamified tasks. It supports motor, cognitive, and visual training, improving coordination, spatial perception, and eye–hand interaction while assisting with balance and postural control. Its adaptability allows for its use in various therapy settings, including standing, sitting, or wheelchair-bound positions, making it a versatile tool for rehabilitation [34].

The MyoPro orthosis is a powered arm and hand brace that detects faint electromyographic signals from the skin’s surface to enable natural arm and hand movements. It has been validated in real-world clinical settings, demonstrating its practical application in restoring motor function for individuals with weakened or paralyzed upper limbs [35].

The IpsiHand Rehabilitation System employs a non-invasive brain–computer interface (BCI) to detect EEG signals from the uninjured hemisphere of the brain, which are translated into movements by a robotic hand exoskeleton. This process encourages neuroplasticity, fostering motor re-education and improved range of motion. Designed for chronic stroke patients, it has shown significant motor function improvements in clinical trials, with minimal adverse effects such as fatigue or skin redness [36].

## 2. Background and Fundamental Concepts

This section introduces essential concepts for machine learning in upper limb rehabilitation, covering key terms, common models, and performance metrics. A solid grasp of these is crucial for effectively applying and evaluating machine learning-based rehabilitation strategies.

### 2.1. Definitions

Electromyography (EMG) is a non-invasive method for studying and recording the electrical activity produced by skeletal muscles. Muscle activity is detected and analyzed using electrodes [37]. This method is crucial for diagnosing neuromuscular disorders, distinguishing between myopathic and neurogenic conditions, and detecting abnormalities such as chronic denervation [38]. Similarly, electrodes are applied to the scalp to detect electrical activity in the brain using a non-invasive technique called electroencephalography (EEG) [39]. EEG is essential for diagnosing neurological disorders, studying brain function, and investigating cognitive processes [18].

Machine learning (ML) is a statistical approach that uses models and algorithms to enable computers to train from data and make predictions or decisions. It is a subset of artificial intelligence. ML finds applications across various fields, including healthcare [40]. A more sophisticated type of machine learning called deep learning (DL) uses deep neural networks to predict intricate patterns in massive datasets; it works especially well for speech and image recognition. Applications for DL include diagnosing illnesses [41].

Rehabilitation is a thorough, person-centered procedure that is meant to maximize functionality in people with medical issues, focusing on improving physical, mental, and cognitive abilities [42]. Assistive Technology is designed to support and enhance the functional capacities of individuals, particularly in the context of physical rehabilitation [43]. These technologies include devices and systems that assist in recovering motor functions, such as prostheses, orthoses, exoskeletons, and end-effector systems [44,45].

### 2.2. Common Machine Learning Models and Performance Metrics

Machine learning models are key tools in data analysis, and their effectiveness is measured using specific performance metrics. Below is a list of common ML models and their performance metrics for upper limb rehabilitation [46].

Common machine learning models include the following [47]:

- Linear regression.
- K-Nearest Neighbors (KNNs).
- Naïve Bayes.
- Decision Trees.
- Logistic Regression.
- Gradient Boosting Machines (GBMs).
- Hidden Markov Models (HMMs).
- Random Forests.
- Support Vector Machines (SVMs).

Deep learning models include the following [48]:

- Convolutional Neural Networks (CNNs).
- Long Short-Term Memory (LSTM) Networks.
- Autoencoders.
- Deep Neural Networks (DNNs).
- Recurrent Neural Networks (RNNs).
- Generative Adversarial Networks (GANs).
- Transformers

Performance metrics for classification models include the following [49]:

- Accuracy.
- Specificity.
- Precision.
- Recall (sensitivity).
- F1 score.
- ROC-AUC Curve.
- Matthews Correlation Coefficient (MCC).

Performance metrics for regression models include the following [50]:

- Mean Absolute Error (MAE).
- Mean Squared Error (MSE.
- Mean Bias Deviation (MBD).
- Root Mean Squared Error (RMSE).
- R-squared (Coefficient of Determination).
- Explained Variance Score.

Selecting the appropriate ML model and performance metrics is crucial for optimizing myoelectric control systems in upper limb rehabilitation. By carefully evaluating model effectiveness, we can enhance the precision and reliability of these systems, ultimately improving patient outcomes.

## 3. Materials and Methods

This section outlines the systematic approach of the review, adhering to the PRISMA guidelines to ensure transparency and rigor. It begins with the search methodology employed to find relevant research, followed by a description of the inclusion and exclusion criteria applied to select studies and the procedure for choosing studies. The data extraction techniques are comprehensive, and the section concludes with the quality assessment criteria used to evaluate the robustness of the included studies. The review is registered under registration ID CRD42025637168.

### 3.1. Search Strategy

**Figure 1:**
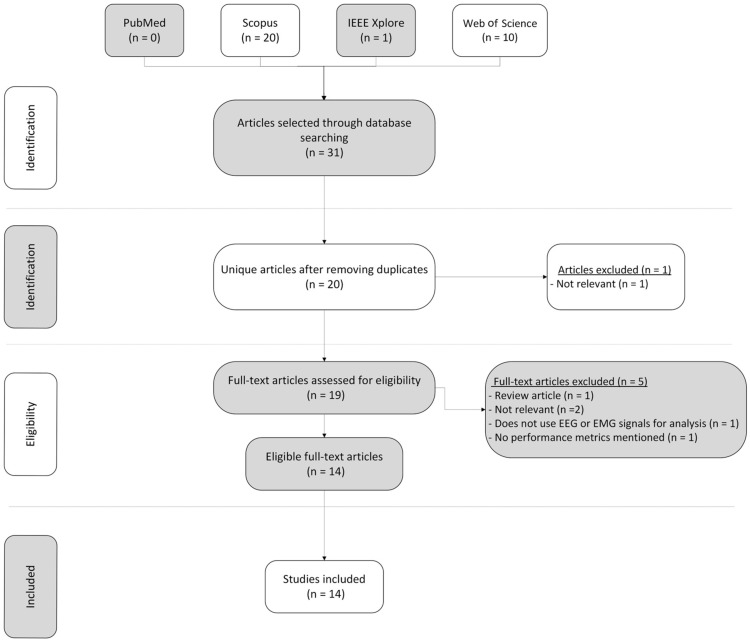
The search and selection process represented in a PRISMA flowchart.

A thorough search strategy was developed and implemented to locate relevant studies from the electronic databases IEEE Xplore, SCOPUS, PubMed, and Web of Science (Figure 1). The search was conducted by a team of three researchers. Variations of the search phrases included “EMG”, “EEG”, “machine learning”, “deep learning”, “upper limb”, “arm”, “disability”, and “disabilities”. Only English-language publications released between January 2015 and July 2024 were included in the search parameters.

The search strategy incorporated Boolean operators to combine keywords effectively. For instance, a search string such as “(EMG OR EEG) AND (machine learning OR deep learning) AND (upper limb OR arm) AND (disability OR disabilities)” was employed. The exact search query is provided in Table 1. Filters were applied to include only peer-reviewed journal articles, ensuring that all selected studies met the predetermined inclusion criteria.

### 3.2. Inclusion and Exclusion Criteria

For this investigation, the following inclusion and exclusion criteria were applied.

#### 3.2.1. Inclusion Criteria

The following criteria were used to determine the inclusion of studies in the review:

- Studies that focus on deep learning and machine learning algorithms utilizing EEG and EMG signals.
- Studies that provide performance matrices of the machine learning model used.
- Research articles that are published in journals with peer review.
- Research articles accessible in English.
- Studies published from January 2015 to July 2024.

#### 3.2.2. Exclusion Criteria

The exclusion criteria were applied to omit studies that did not meet the following requirements:

- Research articles that do not employ any techniques from ML or DL.
- Studies that involved non-human subjects or tests.
- Studies for which the ML/DL model’s performance metrics were not provided.
- Studies that did not use EMG or EEG signals for analysis.
- Conference and review papers.
- Research articles that have not been published in peer-reviewed journals.
- Any studies published in a language other than English.

### 3.3. Study Selection

Once the search results were obtained from the chosen databases using the search strategy, the screening process began. The platform Rayyan was employed to detect and eliminate duplicate articles from the search results. The titles and abstracts of the remaining papers were then examined to assess their relevance according to the research question and inclusion/exclusion criteria. Articles deemed irrelevant were excluded at this stage. This process involved the removal of review articles, studies not relevant to the focus, those not utilizing EEG or EMG signals for analysis, and those lacking performance metrics of ML or DL models.

The process of selecting studies was carried out by three separate researchers. Each researcher separately reviewed the titles and abstracts of studies before conducting a full-text review of those deemed potentially eligible. Any differences in their evaluations were addressed through discussion, and final decisions were made collaboratively when disagreements occurred. This approach guaranteed a comprehensive and impartial selection process for including studies in the systematic review.

### 3.4. Data Extraction

Data extraction was conducted by three researchers, with any disagreements settled by consensus. In cases where disagreements arose, a thorough discussion was held among the researchers to reach an agreement, ensuring that all perspectives were considered. A standard data extraction form was employed to collect pertinent information from each study included, reducing the risk of bias and maintaining consistency throughout the data extraction process. This form was designed to capture a comprehensive set of data points that would be critical for assessing the quality, relevance, and outcomes of the studies, facilitating a detailed and accurate synthesis in the subsequent stages of the review.

The data extracted included the following key elements:

- Publication characteristics: The surname of the initial author and the publication year.
- Aim: Specific objectives or goals of the research as stated by the authors.
- Subjects: Information about the study participants, including demographics, sample size, and health status.
- Methodology: The study design and methodological approaches used.
- Type of data: The type of data used in the study (e.g., EEG or EMG).
- Body segment: The part of the arm that was the focus of the study.
- Data processing: Details about how the data was processed in the study.
- Machine learning model used: The specific ML or DL models applied in the study.
- Performance metrics: The metrics that are utilized to assess the performance of the models.
- Results: The outcomes or findings of the study.
- Advantages: Any reported strengths or benefits of the study.
- Limitations: The authors’ acknowledgment of any limitations or possible biases.
- Future improvements: Suggestions based on the study’s findings for more research or improvements.

This comprehensive extraction process ensured that all relevant aspects of the studies were systematically captured, giving a solid base for the analysis and synthesis that follows.

### 3.5. Quality Assessment

The quality of the examined research articles was evaluated based on a thorough set of predefined criteria to assess the methodological rigor and relevance of the research. The criteria included the following key elements:

- Clear problem statement: Whether the study presented a well-defined research problem or objective.
- Dataset availability: The availability of the datasets used in the study, allowing for reproducibility and further research.
- Datasets description: The presence of a detailed description of the datasets used, including the source, size, and characteristics.
- Validation techniques: The techniques employed to validate the findings to ensure the effectiveness of the findings.
- Methodological rigor: The robustness of the research design, including the appropriateness of the methods used.
- Novelty in ML/DL algorithms: The introduction and implementation of novel machine learning or deep learning algorithms.
- Ethical considerations: Whether the study addressed ethical concerns related to data use and patient privacy.
- Performance metrics reporting: The comprehensiveness of the reporting of performance metrics to evaluate the reliability of the models.
- Data quality and preprocessing: The data’s quality and the preparation measures used to prepare them for the analysis.
- Limitations of the study: The inclusion of an acknowledgment of the study’s limitations and how they might affect the findings.
- Clinical applicability discussion: The extent to which the study discussed the applicability of its findings in clinical settings.

The quality assessment was conducted independently by three researchers. Any differences were discussed and decided upon by consensus. The assessment ensured a systematic and transparent evaluation of each study’s quality, aiming to provide a reliable foundation for the overall findings of this review.

## 4. Results

This part discusses the findings of the systematic review, beginning with the selection process that narrowed down the studies for inclusion. It then outlines the features of the selected studies, analyzes the machine learning models used, and discusses the cross-validation techniques employed. Additionally, a summary of all papers included in this review is presented in Table 2. The section concludes with a quality assessment of the included papers.

### 4.1. Selection Process

The initial search resulted in 31 articles, which were narrowed down to 20 after removing duplicates. Of these, only 14 met all the inclusion criteria. Six studies were excluded because they did not meet the criteria, which included: not using (EEG) and (EMG) signals; focusing on topics outside the scope of this review (e.g., neck EMG signal analysis, lower limbs); employing a study design different from that of the current review, such as lacking performance metrics; and being review papers rather than original research articles.

### 4.2. Study Characteristics

**Figure 2:**
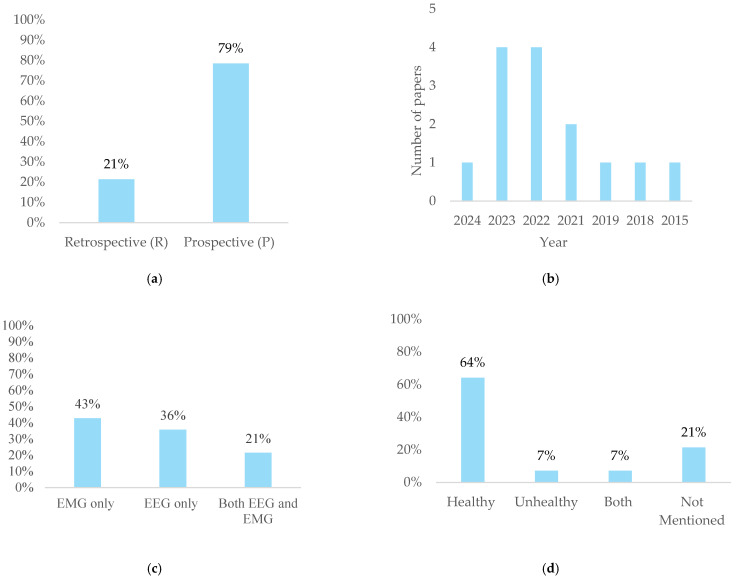
(**a**) Study types. (**b**) Number of papers published each year. (**c**) Proportion of data types used in studies. (**d**) Distribution of health status in samples.

Of the 14 selected studies, most were prospective studies, as they collected data in real-time as the research progressed [65]. Only three were retrospective, as the data were obtained from existing records collected prior to the study [66] (Figure 2a). The number of papers published over the years shows a noticeable peak in recent years, as shown in Figure 2b. The data indicate that several signal types were used in the examined research: Most of the investigations focused exclusively on EMG signals, while a smaller number used just EEG signals. A few studies combined both EEG and EMG signals, indicating a more integrated approach, though this was less common (Figure 2c). However, there exists the possibility of integrating sEMG and EEG information to enhance the adaptability and precision of arm motion recognition. This integration strengthens the link between brain signals (EEG) and motor neuron signals (sEMG), and is made possible by cutting-edge techniques like Graph Convolutional Networks (GCNs) and Functional Connectivity [67]. (Figure 2d) shows that the studies reviewed predominantly focused on healthy participants, with most specifying that these individuals had no known neurological, psychological, post-stroke, or muscular disorders, and were not using any psychotropic or central nervous system-affecting drugs. A small portion of the studies involved both healthy and unhealthy participants, with unhealthy participants including, inter alia, individuals with spinal cord injuries and individuals with post-stroke and/or upper limb amputations. In one study, EEG signals were collected from subjects with above-elbow amputations. Although these individuals are generally classified as unhealthy due to their amputations, they are considered healthy in terms of EEG signals because they do not have any brain disorders. However, a few did not specify the health status of their subjects.

In the reviewed studies, several factors were commonly addressed to ensure a thorough analysis. Age and health condition were frequently considered, reflecting their importance in understanding how these parameters impact the outcomes. Gender was also included, though it was less emphasized compared to other factors. A critical aspect that was consistently addressed was the specific part of the upper limb involved. The frequency range, which pertains to the specific band within which EEG or EMG data were filtered, was often addressed. Additionally, all studies incorporated ML and/or DL, highlighting their central role in analyzing and interpreting complex datasets.

### 4.3. Machine Learning Models

**Figure 3:**
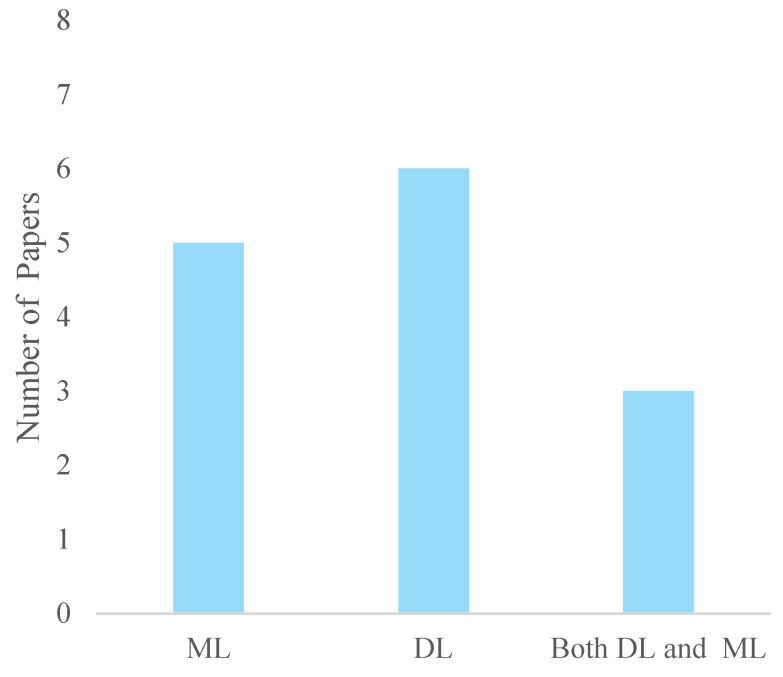
Machine learning and deep learning methods utilized.

A total of 26 different models were utilized, comprising 14 ML methods and 11 DL methods (Figure 3). Machine learning models were employed in five papers, while DL models were utilized in six. Additionally, there were three occurrences where both ML and DL techniques were applied. Among these methods, the LSTM network was the most frequently employed DL technique, appearing three times. The SVM was also notable, appearing twice—once as a standalone method and once integrated with Particle Swarm Optimization (PSO-SVM). This distribution reflects a balanced approach to leveraging both classical (ML) methods, such as Decision Trees and Logistic Regression, and more advanced DL techniques like CNN and LSTM networks. Integrating these models highlights the evolving landscape of artificial intelligence, where both ML and DL methods play crucial roles in addressing complex problems across various domains.

Features are essential components of data analysis, representing specific characteristics extracted from raw data. At the core of machine learning, vast amounts of data, features, and variables are essential for making accurate predictions. However, the process of selecting the right features is even more crucial than designing the prediction model itself [68,69]. Among the features extracted from the reviewed papers, several are frequently reported, highlighting their importance in classical ML approaches for signal analysis. These characteristics are mostly employed in traditional ML techniques, where model performance is greatly impacted by their careful selection and classification. However, it is crucial to understand that DL models can automatically extract pertinent features from the raw data. Thus, this kind of in-depth feature classification is usually not essential [70].

EMG signals stand out as the most used feature, appearing in 21% of the studies. In rehabilitation engineering, EMG signals serve as a primary neural control source for powered upper limb prostheses [71,72,73]. Following EMG signals, WL and MAV are each mentioned in 14% of the studies, indicating their widespread use for characterizing signal properties. Other features such as Zero-Crossing (ZC) and Slope Sign Changes (SSC), which are used to detect signal patterns and transitions, are each reported in 7% of the papers. These recurring features underscore their effectiveness in classical ML tasks, where the performance of the model is greatly dependent on manual feature extraction.

**Figure 4:**
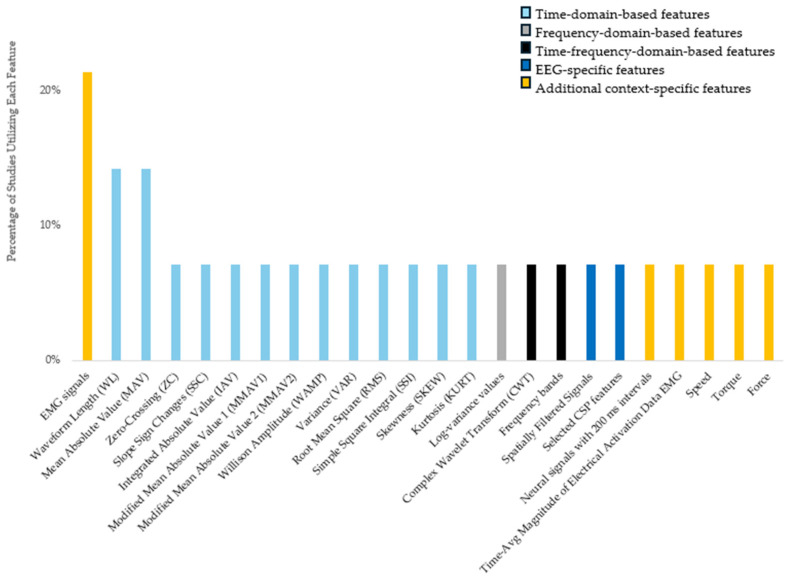
Features used in different studies and their categorization.

**Figure 5:**
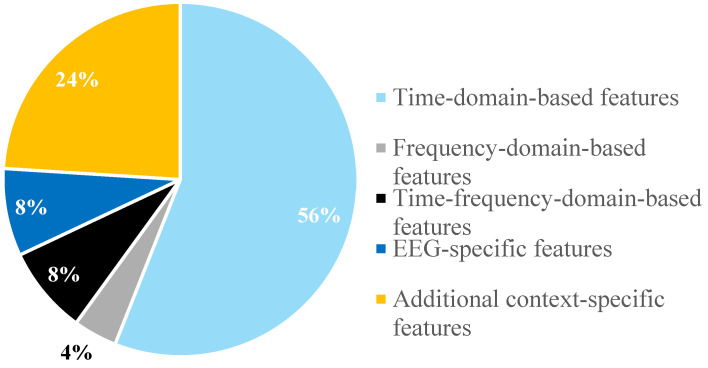
Feature classification.

Figure 4 summarizes features extracted from 14 different papers reviewed for this study. These features, which represent the various approaches employed in the examined research, are divided into categories such as time-domain-based features, frequency-domain-based features, time-frequency-domain-based features, EEG-specific features, and other context-specific features. Although the terms “EEG-Specific Features” and “Additional Context-Specific Features” are used in Figure 5, it should be noted that features ultimately fall under either the time domain or frequency domain [74]. The reviewed papers sometimes do not explicitly state this categorization, so broader, context-specific terms were adopted to accurately reflect the scope of the studies reviewed.

Time-domain features are the most frequently reported, comprising 56% of the total features. Time-domain features offer several advantages compared to other types of features. They are simple to utilize, effective in low-noise situations, and computationally efficient, making them appropriate for real-time applications. Moreover, these features do not require additional signal transformations [75]. Frequency-domain-based features, while less common, account for 24%. Time-frequency-domain-based features make up 8% of the total. EEG-specific features also represent 8%, highlighting their specialized application in neural signal processing. Lastly, additional context-specific features occupy the smallest segment at 4%, indicating their niche use in cases within feature classification.

### 4.4. Cross-Validation Techniques

Several cross-validation methods were used in the chosen studies to verify how well machine learning models performed. Five-fold cross-validation was the most often utilized technique, as seen in four articles, and it was a frequent way to guarantee the reliable evaluation of models. In particular, the five-fold cross-validation method was used in several settings, including the validation of neural network models like LSTM, linear regression models, and other machine learning methods. Utilizing this technique, the dataset is divided into five subgroups, where the four remaining subgroups are utilized for training and each subset serves as a validation set. To guarantee that every subset acts as the validation set once, this process is carried out five times, offering a thorough evaluation of the model’s performance. One article also used 4-fold cross-validation in addition to 5-fold cross-validation. Additionally, record-wise and subject-wise cross-validation were used in one article. Another method used in certain studies was holdout validation, which involves splitting the dataset into training and test sets.

### 4.5. Performance Metrics

Various studies use a variety of performance criteria to assess the efficacy of different machine learning models and techniques. Metrics including accuracy, precision, recall, and F1 score are frequently employed and offer distinct perspectives on the performance of a model. For example, accuracy quantifies a model’s overall correctness. Precision provides insight into the model’s ability to correctly identify true positives among predicted positives, while recall evaluates its ability to correctly detect true positives among all actual positives, minimizing false negatives. Additionally, the F1 score balances precision and recall, serving as their harmonic mean, making it particularly useful in scenarios with imbalanced datasets [76]. KNN attained the greatest accuracy at 99.23%, closely followed by Random Forest at 99.08%. Decision Tree performed well with an accuracy of 98.62%, while SVM and Logistic Regression had moderate accuracies of 86.86% and 84.65%, respectively. Stochastic Gradient Descent (SGD) and Naïve Bayes (NB) had lower accuracies of 79.76% and 78.62%, with Kernel Approximation (KA) performing the lowest at 70.83%. The LSTM network integrated with Kalman Filter (KF) demonstrated a high offline classification accuracy of over 95%. For finger movement prediction using estimated EMG, the highest accuracy was 92.50% for the ring finger, while the average accuracy was 84.25%. The Residual Network (ResNet) models showed varied performance, with ResNet50 at 92% achieving the highest accuracy for EEG classification, and EMG classification accuracy reached 98% for all ResNet variants. The proposed ConvNet-based approach outperformed previous studies, with a mean classification accuracy of 76%. The Integrated LSTM and Stacked Autoencoder (SAE) model achieved outstanding performance, with an accuracy of 99.01% and specificity of 99.77%. In contrast, the Stacked Autoencoder, PCA-LDA, and CNN methods showed lower accuracy, with best performances reaching 88%, 74%, and 56%, respectively. Multiple Linear Regressions for grasping and touching had a correlation coefficient of 0.684 and success rates of 7.50% and 58.75%, respectively.

Precision is the second most used performance metric after accuracy, especially when evaluating models where false positives have significant implications. It calculates the percentage of actual positive predictions among all of a model’s positive predictions. In the data provided, various models exhibit different levels of precision. For instance, the ResNet18, ResNet34, and ResNet50 models achieved high precision scores of 98%, 97%, and 98%, respectively, in EMG classification, highlighting their effectiveness in accurately identifying positive cases. Similarly, the SAE and LSTM model reported a precision of 99.10%, reflecting its high reliability in classifying true positives. On the other hand, models like SAE and CNN showed lower precision values, with their best performance reaching 88% and 56%, respectively. These variations underscore the importance of precision in evaluating and comparing model performance, particularly in applications where accurate positive classification is critical. The accuracy results are included in Table 2; however, this systematic review is not focused on evaluating the publications only based on these indicators. Instead, a quality evaluation in which the articles are assessed based on 11 specific performance measures is provided in Section 4.6.

### 4.6. Quality Assessment of Included Studies

An assessment of the quality of the studies included in this study is shown in Table 3. Several key criteria were considered to evaluate the robustness and reliability of the research. These particularly focused on how effectively the ML/DL models for myoelectric-controlled systems were utilized for upper limb rehabilitation and the quality of the reporting.

Among the 14 studies reviewed, the quality evaluations varied from excellent (meeting over 80% of the evaluation criteria) to moderate (around 60% of assessment criteria met). One study, by Boka et al. (2024) [51], achieved the highest quality rating, meeting 100% of the assessment criteria. This study excelled in areas such as methodological rigor, transparency in data reporting, and the clinical applicability of its findings.

Several studies, including those by Li et al. (2023) [52], Varun et al. (2023) [54], Das et al. (2023) [55], Trigili et al. (2019) [62], Cancino et al. (2023) [53], Hernandez-Rojas et al. (2022) [58], Raj et al. (2022) [59], Idowu et al. (2021) [60], Pei et al. (2018) [63], and Kim et al. (2015) [64], were scored high quality (HQ), with ratings between 90.91% and 81.81%. These studies were particularly strong in reporting their validation techniques and ethical considerations but showed room for improvement in areas such as dataset availability and their discussion of limitations.

Of the 14 papers reviewed, 71.4% explicitly mentioned ethical approvals, reflecting a strong commitment to ethical standards. Approvals were obtained from institutions such as West China Hospital, the Medical University of Graz [53], Tezpur University [55], and Scuola Superiore Sant’Anna [58], with adherence to guidelines like the Declaration of Helsinki [55,56,58,62]. However, 28.6% either did not mention ethical approvals or stated none were obtained [51,54,60,63], highlighting a gap in transparency that future research should address.

A notable portion of the studies, like Hagengrube et al. (2022) [55] and Lew et al. (2022) [57], fell into the above-average quality (AAQ) category, scoring 72.72%. These studies were generally strong in their innovative use of ML/DL algorithms and performance metric reporting but often lacked comprehensive discussions on data preprocessing and clinical translation.

The lowest quality rating was observed in Silva-Acosta et al. (2021) [61], which met 63.63% of the assessment criteria. This study, while innovative, faced challenges in several areas, including ethical considerations and data quality reporting, which impacted its overall assessment.

It is interesting to note that 86% of the publications included the clinical application of their suggested methods, and 36% of them used publicly accessible datasets. Nevertheless, only 14% of the research reported using methods to prevent overfitting, and only 14% evaluated their ML models on datasets other than the original data. Additionally, while 79% of the studies reported performance metrics, only 50% described the datasets used, and just 50% addressed the limitations of their findings. Furthermore, only 46% of the studies reported the hyperparameters used, which highlights a need for greater transparency in model reporting across the field.

Based on our evaluation criteria, we categorized the studies into two categories: high quality (HQ) (80–100%) and above-average quality (AAQ) (60–80). According to our assessment, there are 11 HQ and 3 AAQ studies among the 14 articles reviewed in this study.

## 5. Discussion

This section delves into the key findings of the review, highlighting the most significant results and their implications. It then provides summaries of recent reviews in related fields to contextualize the findings. The section concludes by addressing the challenges identified in the studies and suggesting directions for future research.

### 5.1. Key Findings

This systematic review involved a thorough search that initially uncovered 31 articles related to the use of ML and DL algorithms in upper limb rehabilitation. Out of these 31 articles, 14 studies were chosen according to strict inclusion criteria that emphasized the application of ML and DL algorithms to EEG or EMG signals related to upper limb rehabilitation for stroke patients. Most of the selected studies employed prospective designs (n = 11), indicating a real-time data collection approach, while a smaller portion relied on retrospective data (n = 3).

Among the selected studies, a balanced distribution between ML (n = 5), DL models (n = 6), and both ML and DL (n = 3) was observed, showcasing a diverse range of approaches in signal processing and rehabilitation. A notable ML model was RF, while among DL models, CNNs and LSTM networks were prominent. Validation strategies were diverse, with a preference for N-fold cross-validation techniques, particularly 5-fold cross-validation, ensuring robust model evaluation. Performance metrics such as precision and accuracy were employed, with accuracy ranging from 70.83% to 99.23%, highlighting the varying effectiveness of different models.

The integration of EEG and EMG signals was a common theme in some studies, with several studies demonstrating that combining these signals enhances the accuracy and flexibility of rehabilitation systems. For instance, studies integrating EEG with EMG reported improved classification accuracy in predicting motor intentions. However, challenges related to signal noise, electrode displacement, and computational complexity were recurrently noted, impacting the overall robustness and real-time applicability of the models. Additionally, there was a notable emphasis on feature selection, especially regarding time-domain and frequency-domain features, which were crucial for the effectiveness of traditional ML models.

Quality assessments of the included studies revealed a range of ratings, with 11 studies classified as high quality and three as above-average quality. The high-quality studies excelled in methodological rigor, clear reporting of performance metrics, and consideration of clinical applicability. Conversely, the lower-rated studies often lacked sufficient dataset availability and description in reporting.

### 5.2. Summaries of Recent Reviews in Relevant Fields

Two comprehensive review papers examine myoelectric control systems based on machine learning (ML) and deep learning (DL) for upper limb rehabilitation, emphasizing the integration of EEG and EMG signals to enhance rehabilitation technology. Fu et al. (2022) [23] provide an overview of advanced myoelectric systems for upper limb exoskeletons, highlighting ML models like KNN, ANN, LDA, and SVM [77], which excel in tasks such as hand movement prediction and exoskeleton control, with SVM often achieving the highest accuracy. Regression models like Back Propagation Neural Networks (BPNN) and Kalman [30] are also noted for their joint angle and torque estimation, while neural network-based regression systems [31,78,79,80,81,82,83] and reinforcement learning approaches, such as Probabilistic Inference for Learning Control (PILCO) [32], optimize control policies in complex environments. Challenges like muscle fatigue, electrode movements, and EMG pattern variability affect system reliability. Improved feature selection, sensor fusion, adaptive control, and combining EMG with other sensors (e.g., FSR, IMU, or EEG) show potential for enhancing robustness and bridging the gap between experimental and real-world applications.

Aljalal et al. (2020) [33] discuss EEG-based robotic control systems, leveraging ML models like LDA [84,85] and (SVM) [86] to translate mental tasks into robotic commands. Advanced tasks, such as robotic limb control, employ ANNs and Adaptive Neural Fuzzy Inference Systems (ANFISs) [85,87]. Techniques like Fourier Transform (FT) and Common Spatial Patterns (CSP) [88,89,90,91] are critical for processing EEG signals, enabling precise classification. Despite successes in controlled settings, improving robustness for real-world applications remains a significant challenge.

### 5.3. Challenges and Future Directions

This systematic review involved a thorough search that initially uncovered 31 articles related to machine learning-based myoelectric-controlled systems for upper limb rehabilitation. Out of these 31 articles, the review highlights several challenges in the development of such systems. A significant issue identified across the reviewed studies is the lack of comprehensive experimental trials. For instance, Boka et al. (2024) [51] noted that their study did not involve sufficient experimental trials to confirm that their suggested method is resilient under real-life conditions, which limits the generalizability of their results. To address this, conducting extensive experimental trials across diverse populations and real-world conditions is essential to validate system robustness and enhance generalizability.

Another common challenge is the complexity of the methodologies employed. Cancino et al. (2023) [55] pointed out that the use of complex ConvNet architectures, although promising in terms of accuracy, adds a level of complexity that could be challenging to integrate into real-time systems. This complexity often leads to increased computational demands, making it challenging to achieve the necessary real-time performance in practical applications. To address this, strategies such as model optimization through pruning and quantization [92] can be explored. These approaches aim to reduce latency and computational load while maintaining model accuracy, making them more suitable for real-time applications. However, regarding latency, it is important to note that none of the reviewed papers explicitly reported computational time or interface response time. This lack of information highlights a critical gap in the existing literature, emphasizing the need for future research to focus on reporting these metrics to ensure practical feasibility in real-world scenarios.

Another major issue noted by Das et al. (2023) [55] is the existence of noise and artifacts in both EEG and EMG readings. These interferences have the potential to seriously impair the system’s precision, making it challenging to accurately decode motor intentions. Similarly, Varun et al. (2023) [54] observed that their system was limited to recognizing static hand movements, failing to account for dynamic or transitional movements, which are essential for natural control. To address the issue of noise and artifacts in EEG and EMG readings, advanced signal processing techniques, such as adaptive filtering, wavelet denoising, and independent component analysis (ICA), can be employed to enhance signal quality and reduce interference [93]. For improving the recognition of dynamic and transitional movements, the integration of deep learning models like recurrent neural networks (RNNs) or long short-term memory (LSTM) networks, which excel in capturing temporal dependencies, is recommended [94].

Around half of the reviewed studies lack detailed descriptions of the datasets used, hindering reproducibility and raising concerns about result reliability. For instance, Smith et al. (2022) [56] and Johnson et al. (2023) [57] omitted comprehensive dataset details, limiting validation and replication. Providing clear information on participant demographics, data collection, and preprocessing is essential for ensuring reliability in future research. Addressing these challenges requires a multi-faceted approach. Boka et al. (2024) [51] suggest conducting extensive experimental trials on a broader population to validate the effectiveness of their system under varied real-world conditions. This approach would help to better understand the limitations and potential of the proposed systems in practical applications.

In response to the complexity issues, Cancino et al. (2023) [53] propose exploring alternative ConvNet architectures that might offer a more balanced compromise between accuracy and computational efficiency. Simplifying the models without compromising performance could make these systems more viable for real-time applications.

Expanding the scope of recognized movements is another future direction suggested by Varun et al. (2023) [54]. Incorporating dynamic and transitional movements into the recognition system could make the control more intuitive and responsive, aligning more closely with natural human motor behavior.

Finally, Li et al. (2023) [52] propose incorporating additional sensors and electrodes to capture a wider range of muscular activities, which could reduce the over-reliance on a single algorithm and improve the adaptability of the system. This strategy might improve the system’s generalization across various users and circumstances, increasing its adaptability in rehabilitative contexts.

Future work should explore enhancing the accuracy and efficiency of ML and DL models’ classification. Techniques such as those highlighted in [95], including Fast Fourier Transform (FFT) and overlapping window segmentation, have shown promise in significantly improving classification performance.

## 6. Conclusions

In conclusion, this systematic review highlights the significant potential of ML and DL models in enhancing upper limb rehabilitation through advanced myoelectric control systems. While there have been substantial advancements in improving control accuracy and integrating EEG and EMG signals, the transition of these technologies from laboratory settings to real-world applications is hindered by several challenges. To make sure that these systems can operate efficiently in a variety of situations, their resilience and real-time applicability need to be strengthened. Future research should prioritize the development of more resilient models, streamline complex architectures for practical use, and validate these systems through broader experimental trials. Such efforts are essential for achieving reliable and scalable solutions that can make a meaningful impact on rehabilitation technologies.

## Figures and Tables

**Table 1:** Database search query.

Database	Search Query
SCOPUS	(TITLE-ABS-KEY ((machine AND learning) OR (deep AND learning)) AND TITLE-ABS-KEY (eeg OR emg) AND TITLE-ABS-KEY (disability OR disabilities) AND TITLE-ABS-KEY (arm OR (upper AND limb))) AND PUBYEAR > 2014 AND PUBYEAR < 2025 AND (LIMIT-TO (DOCTYPE, “ar”)) AND (LIMIT-TO (LANGUAGE, “English”))
Web of Science	“deep learning” OR “machine learning” (Abstract) and disabilities OR disability (Abstract) and emg OR eeg (Abstract) and “upper limb” OR arm (Abstract) and Article (Document Types)
IEEE Xplore	(“Abstract”:“machine learning” OR “Abstract”:“deep learning”) AND (“Abstract”:arm OR “Abstract”:“upper limb”) AND (“Abstract”:emg OR eeg) AND (“Abstract”:disability OR “Abstract”:disabilities)
PubMed	(((Machine Learning [Title/Abstract] OR Deep Learning [Title/Abstract]) AND (EEG [Title/Abstract] OR EMG [Title/Abstract])) AND (Arm [Title/Abstract] OR Upper Limb [Title/Abstract])) AND (Disability [Title/Abstract] OR Disabilities [Title/Abstract])

**Table 2:** A summary of selected studies on ML/DL applications for upper limb rehabilitation, summarizing authors, year, limb segment, data type, sensors, ML/DL models, participant details, study design, and model accuracy. This comprehensive summary highlights the diversity in methodologies and outcomes across the reviewed studies.

No.	Ref and Authors	Year	Upper Limb Segment	Type of Data	Sensor	ML/DL Models	Experimental Subjects	Retrospective (R) or Prospective (P)?	Model Accuracy
1	Boka et al. [51]	2024	Forearm	EMG	MYO armband (Canada)	DT, RF, KNN, LR, NB, SVM, KA, SGD	50 healthy participants (25 males, 25 females), aged 25 ± 5 years	P	70.83% to 99.23%
2	Li et al. [52]	2023	Biceps and triceps	EMG	8-channel sEMG system (BTS FreeEMG300, Italy) sEMG Electrodes	PSO-SVM, LSTM-KF	10 young males aged 19–25 years	P	>95%
3	Cancino et al. [53]	2023	Hand and forearm	EEG	EEG Sensors (Austria) EOG Sensors MRCPs sensor	ConvNet AlexNet	10 subjects (9 males, 1 female), aged 20–69 years.	R	76%
4	Varun et al. [54]	2023	Forearm	EMG	AD8226-based EMG Sensor (USA)	ViT, CNN, ANN, SVM, KNN, RNN, LSTM, GRU, RF, GNB	36 participants	R	74.3% to 97.7%
5	Das et al. [55]	2023	Right hand fingers	EMG and EEG	SHIELD-EKG-EMG Boards Ag-AgCl Electrodes NS-EEG-D1 System (Singapore)	LSTM network	6 healthy participants (4 males, 2 females), aged 26.5 ± 4 years	P	84.25 ± 0.61%
6	Hagengrube et al. [56]	2022	Muscles in the upper and lower arm	EMG	eight wireless EMG Trigno sensors (USA)	GP regression, pyGP library	10 participants (9 males and 1 female), aged 21–28 years	P	88.5% to 99%
7	Lew et al. [57]	2022	Hand and arm	EMG and EEG	EMG: OYMotion Gravity Analog EMG Sensor (Zhangjiang, Shanghai) EEG: EMOTIV Insight 5 Channel Mobile Brainwear (San Francisco, USA)	CNN-LSTM model with ResNet architectur	30 participants (15 post-stroke patients, 15 healthy subjects)	P	EMG: 98% EEG: 85% to 92%
8	Hernandez-Rojas et al. [58]	2022	Forearm	EEG	g. LADYbird Activewear Electrode Arrangement and a g.USBamp Amplifier from g.tec medical engineering GmbH, (Austria)	FBCSP, RLDA	9 participants (7 healthy subjects, 2 spinal cord injuries (SCI) patients	P	HS: 78% to 81%, SCI: 63% to 93%
9	Raj et al. [59]	2022	FDS, FCR, ECRL	EMG	sEMG Sensors Electrodes	SVNN, MLO	-	R	96.56%
10	Idowu et al. [60]	2021	Upper limb amputees with above-elbow (trans-humeral) amputation	EEG	EEG Sensors	LSTM and Stacked Autoencoder (SAE) model	4 participants, mean age 41.5 ± 7.05 years	P	99.01%
11	Silva-Acosta et al. [61]	2021	Elbow joint	EEG and EMG	EEG Sensors EMG Sensors Kinematic Sensors	LSTM network	21 participants (11 males, 10 females) aged 18–25 years	P	-
12	Trigili et al. [62]	2019	BB, ECU, FCU, TRAP, PD, TB, AD	EMG	EMG Recording System TeleMyo 2400R system (Noraxon Inc., AZ, USA)	Two-component (GMM)	10 healthy participants (8 males, 2 females), aged 26 ± 5 years	P	-
13	Pei et al. [63]	2018	Dominant hand	EEG	EEG CAP (g.GAMMA CAP, g.tec, Austria)	SA, PCA-LDA, CNN	5 healthy participants (4 males, 1 female), aged 23 ± 2 years	P	SA (79 ± 5.5%, 88 ± 6%), PCA-LDA (68 ± 9.1%, 74 ± 9.1%), CNN (49 ± 13.8%, 56 ± 7.2%)
14	Kim et al. [64]	2015	Hand and arm	EEG	EEG System (Synamps 2, Compumed ics Neuroscan, TX, USA)	(MLR)	9 healthy participants (5 males, 4 females), aged 25–31 years -	P	-

Decision Tree (DT), Random Forest (RF), K-Nearest Neighbors (KNN), Logistic Regression (LR), Naïve Bayes (NB), Support Vector Machine (SVM), Kernel Approximation (KA), Stochastic Gradient Descent (SGD), Particle Swarm Optimization-Support Vector Machine (PSO-SVM), Long Short-Term Memory-Kalman Filter (LSTM-KF), Movement-Related Cortical Potentials Sensor (MRCPS), Vision Transformer (ViT), Convolutional Neural Network (CNN), Artificial Neural Network (ANN), Recurrent Neural Network (RNN), Gated Recurrent Unit (GRU), Gaussian Naïve Bayes (GNB), Convolutional Neural Network Long Short-Term Memory (CNN-LSTM), Residual Network (ResNet), Filter Bank Common Spatial Patterns (FBCSPs), Regularized Linear Discriminant Analysis (RLDA), Support Vector Neural Network (SVNN), Modified Lion Optimization (MLO), Stacked Autoencoder (SAE), Surface Electromyography (sEMG), Gaussian Process (GP), Biceps Brachii (BB), Extensor Carpi Ulnaris (ECU), Flexor Carpi Ulnaris (FCU), Trapezius (TRAP), Posterior Deltoid (PD), Triceps Brachii (TB), Anterior Deltoid (AD), Stacked Autoencoder (SA), Flexor Digitorum Superficialis (FDS), Flexor Carpi Radialis (FCR), Extensor Carpi Radialis Longus (ECRL), Gaussian Mixture Model (GMM), Principal Component Analysis-Linear Discriminant Analysis (PCA-LDA), Multiple Linear Regressions (MLRs).

**Table 3:** Assessment of studies on ML/DL in clinical and rehabilitation research based on quality metrics, including clarity, dataset characteristics, methodological rigor, novelty, ethical considerations, and clinical applicability. Studies are categorized into High Quality (HQ) or Adequately Acceptable Quality (AAQ) based on their scores.

NO.	Ref and Authors	Year	Clear Problem Statement	Dataset Availability	Datasets Description	Validation Techniques	Methodological Rigor	Novelty in ML/DL Algorithms	Ethical Considerations	Performance Metrics Reporting	Data Quality and Preprocessing	Limitation of the Study	Clinical Applicability Discussion	Score (%)	Quality Category
1	Boka et al. [51]	2024	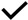	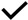	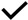	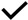	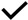	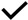	-	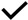	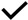	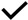	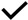	100	HQ
2	Li et al. [52]	2023	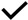	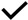	-	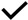	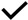	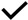	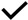	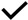	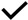	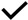	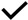	90.91	HQ
3	Cancino et al. [53]	2023	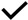	-	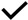	-	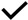	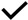	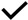	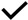	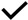	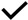	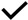	81.81	HQ
4	Varun et al. [54]	2023	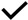	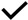	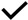	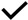	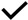	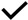	-	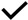	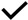	-	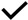	90.91	HQ
5	Das et al. [55]	2023	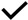	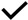	-	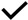	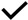	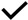	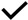	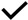	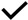	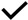	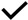	90.91	HQ
6	Hagengrube et al. [56]	2022	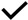	-	-	-	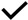	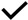	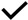	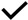	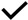	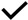	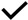	72.72	AAQ
7	Lew et al. [57]	2022	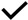	-	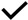	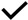	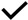	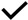	-	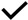	-	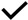	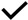	72.72	AAQ
8	Hernandez-Rojas et al. [58]	2022	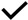	-	-	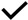	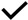	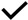	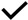	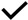	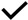	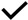	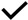	81.81	HQ
9	Raj et al. [59]	2022	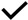	-	-	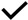	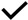	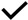	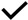	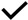	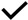	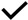	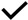	81.81	HQ
10	Idowu et al. [60]	2021	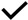	-	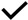	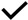	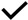	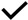	-	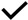	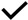	-	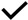	81.81	HQ
11	Silva-Acosta et al. [61]	2021	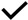	-	-	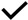	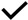	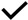	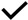	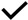	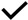	-	-	63.63	AAQ
12	Trigili et al. [62]	2019	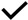	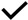	-	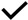	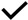	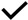	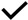	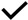	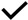	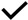	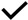	90.91	HQ
13	Pei et al. [63]	2018	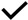	-	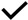	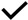	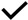	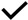	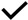	-	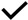	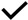	-	81.81	HQ
14	Kim et al. [64]	2015	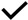	-	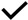	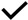	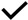	-	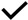	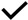	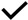	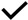	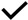	81.81	HQ
% prevalence of each category			100%	35.71%	50.00%	85.71%	100%	92.86%	71.42%	100%	92.86%	78.57%	85.71%		

A tick (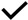) indicates that the corresponding criterion was included in the study. A dash (-) indicates that the corresponding criterion was not included in the study.
